# Quantifying Variation in the Ability of Yeasts to Attract *Drosophila melanogaster*

**DOI:** 10.1371/journal.pone.0075332

**Published:** 2013-09-25

**Authors:** Loida Palanca, Anne C. Gaskett, Catrin S. Günther, Richard D. Newcomb, Matthew R. Goddard

**Affiliations:** 1 School of Biological Sciences, the University of Auckland, Auckland, New Zealand; 2 The New Zealand Institute for Plant & Food Research Limited, Auckland, New Zealand; Ecole Normale Supérieur de Lyon, France

## Abstract

Yeasts that invade and colonise fruit significantly enhance the volatile chemical diversity of this ecosystem. These modified bouquets are thought to be more attractive to *Drosophila* flies than the fruit alone, but the variance of attraction in natural yeast populations is uncharacterised. Here we investigate how a range of yeast isolates affect the attraction of female *D. melanogaster* to fruit in a simple two choice assay comparing yeast to sterile fruit. Of the 43 yeast isolates examined, 33 were attractive and seven repellent to the flies. The results of isolate-versus-isolate comparisons provided the same relative rankings. Attractiveness varied significantly by yeast, with the strongly fermenting *Saccharomyces* species generally being more attractive than the mostly respiring non- *Saccharomyces* species (*P* = 0.0035). Overall the habitat (fruit or other) from which the isolates were directly sampled did not explain attraction (*P* = 0.2352). However, yeasts isolated from fruit associated niches were more attractive than those from non-fruit associated niches (*P* = 0.0188) regardless of taxonomic positioning. These data suggest that while attractiveness is primarily correlated with phylogenetic status, the ability to attract *Drosophila* is a labile trait among yeasts that is potentially associated with those inhabiting fruit ecosystems. Preliminary analysis of the volatiles emitted by four yeast isolates in grape juice show the presence/absence of ethanol and acetic acid were not likely explanations for the observed variation in attraction. These data demonstrate variation among yeasts for their ability to attract *Drosophila* in a pattern that is consistent with the hypothesis that certain yeasts are manipulating fruit odours to mediate interactions with their *Drosophila* dispersal agent.

## Introduction

Saccharomycetes (Hemiascomycete) yeasts are a highly diverse class of microbial fungi that inhabit a variety of niches; however, many can establish and multiply on and in fruits [1]. Whilst rare in the fruit microbial community initially, the *Saccharomyces* *sensu-stricto* species, especially *S. cerevisiae*, typically come to dominant the mature ferments of ripe fruits [2,3]. These *Saccharomyces* species opt for fermentation even in the presence of oxygen (the Crabtree effect), which is the least energetically efficient route to produce energy from the substrate sugars, but it does have a greater rate of ATP production [4]. This strategy, produces ethanol, heat and carbon dioxide, which in combination modifies the fruit niche and excludes other competing microbes from establishing and utilizing the resource [3,5,6]. This modification of the fruit resource to a form that is no longer suited to most other microbes has been described as an example of ecosystem engineering [3]. Another product of yeast manipulation of the fruit niche, which may or may not be linked to fermentation, is the array of odorous volatile chemicals produced [7]. Volatile production consumes ATP and wastes carbon, but other than tyrosol, which is involved in quorum sensing [8], the function of volatile formation and release in yeasts is unknown [9]. Yeasts will typically modify sugars, amino acids and fatty acids to produce esters, higher alcohols, carbonyls, fatty acid derivatives and sulphur compounds, or free volatiles from conjugated forms to release mono-terpenes and thiols [7,10,11]. Different types of yeast vary in their ability to grow in fruits and produce these different volatile compounds. For example, different strains and species of yeast are able to produce different levels of esters and higher alcohols and other volatiles [12-15]. However, it is unknown whether the production of differential complex volatile profiles by yeasts is merely a neutral by-product of other biochemical reactions, or whether this trait that has an active function in the organism’s physiology or ecology [9]. One hypothesis to explain the function of volatile manipulation and production by yeasts is that these odours attract insects, and thus enhance the dispersal and survival of otherwise non-motile yeasts [9].

There is evidence that insects may disperse yeasts, either through ingestion or carrying them externally [16-18]. However, whether volatile production and modification by yeasts in fruits is directly related to enhanced attraction of dispersal agents remains to be thoroughly tested. Vinegar (fruit) flies of the genus *Drosophila* are strong candidates for being yeast dispersers. Many species within this genus use fruit or other decaying plant and fungal material as sites for finding mates and oviposition, and for adult and larval feeding on both the host substrate and its microbial communities [19,20]. There is longstanding evidence of interactions between *Saccharomyces* yeasts and *Drosophila* flies [21-23]. Females of most fruit-associated *Drosophila* species prefer to oviposit on substrates colonized by fermenting yeast [24]. As well as providing essential nutrients, yeasts can make some products more available or less toxic to *Drosophila* , mediate pheromone production [19,23], and influence larval growth, survival and body size [20,23-25]. *S. cerevisiae* and other related yeasts are regularly found in the gut and on the exterior body surfaces of a range of wild caught *Drosophila* species [16,18,22]. Yeasts can also be transferred between courting and mating males and females, and to fruit during oviposition [26]. Much of the interaction between flies and yeast is mediated via the fly’s chemosensory system which through their neurobiology culminates in oviposition behaviour [27,28]. Many of *Drosophila* ’s olfactory receptors are specifically attuned to the esters and higher alcohols that yeasts produce during fermentation [7,9,12,29,30], whereas one receptor is specifically tuned to the compound geosmin produced by many toxic microbes [1,31]. Yeasts produce high levels of carbon dioxide during fermentation and normally this would act as a repellent for *Drosophila* ; however, receptors associated with the detection of carbon dioxide are specifically inhibited in the presence of fruit odours, allowing the flies to find food in a high carbon dioxide environment [2,3,32]. *Drosophila* are also resistant to the alcohol produced during fermentation and have a high frequency of alcohol dehydrogenase alleles conferring alcohol tolerance [4,33,34]. Furthermore, acetic acid is attractive to ovipositing females through their gustatory system, but generally repulsive when detected as an odour [3,5,6,35]. Attractive compounds for *D. melanogaster* include alcohols such as ethanol and 2-phenylethanol, volatile acids, aldehydes such as acetaldehyde, ethyl esters such as ethyl hexanoate, and acetate esters such as ethyl acetate, phenyl ethyl acetate, and isoamyl acetate, most of which are among the array of yeast fruit fermentation volatiles [3,36-38].

Some recent experimental work has shown that *Drosophila* larvae may mediate *Candida* and *Pichia* species densities in fruits [7,39], and that *Drosophila* may stabilise yeast communities in fruits comprising mostly *Candida* *, Pichia, Hansensiapora* and *Saccharomyces* species [8,40]. While it is clear that *Drosophila* and yeasts from varying taxonomic origins may interact, there are few experiments characterising the taxonomic range of yeasts that attract *Drosophila*, and none that have quantified attraction. One study has shown that a combination of esters attract *Drosophila* to *Arum* *palaestinum* (arum lilies), which are thought to mimic fermentation odours [9,41], and another that a blend of five yeast-produced compounds (acetoin, acetic acid, ethanol, 3-methyl-1-butanol and 2-phenyl ethanol) are attractive [7,10,11,24]; however, the odours that correlate with attraction of *Drosophila* to yeast fruit ferments are not known.

Here we investigate how the attraction of *D. melanogaster* to fruit juice varies according to the net result of culturing with a diversity of yeasts isolated from different environments. We found substantial variation in the attraction of flies to different yeasts. Generally we found that fermentative *Saccharomyces* species were the most attractive, but *S. cerevisiae* originating from non-fruit sources were repulsive and some fruit inhabiting non- *Saccharomyces* were highly attractive.

## Results

**Figure 1:**
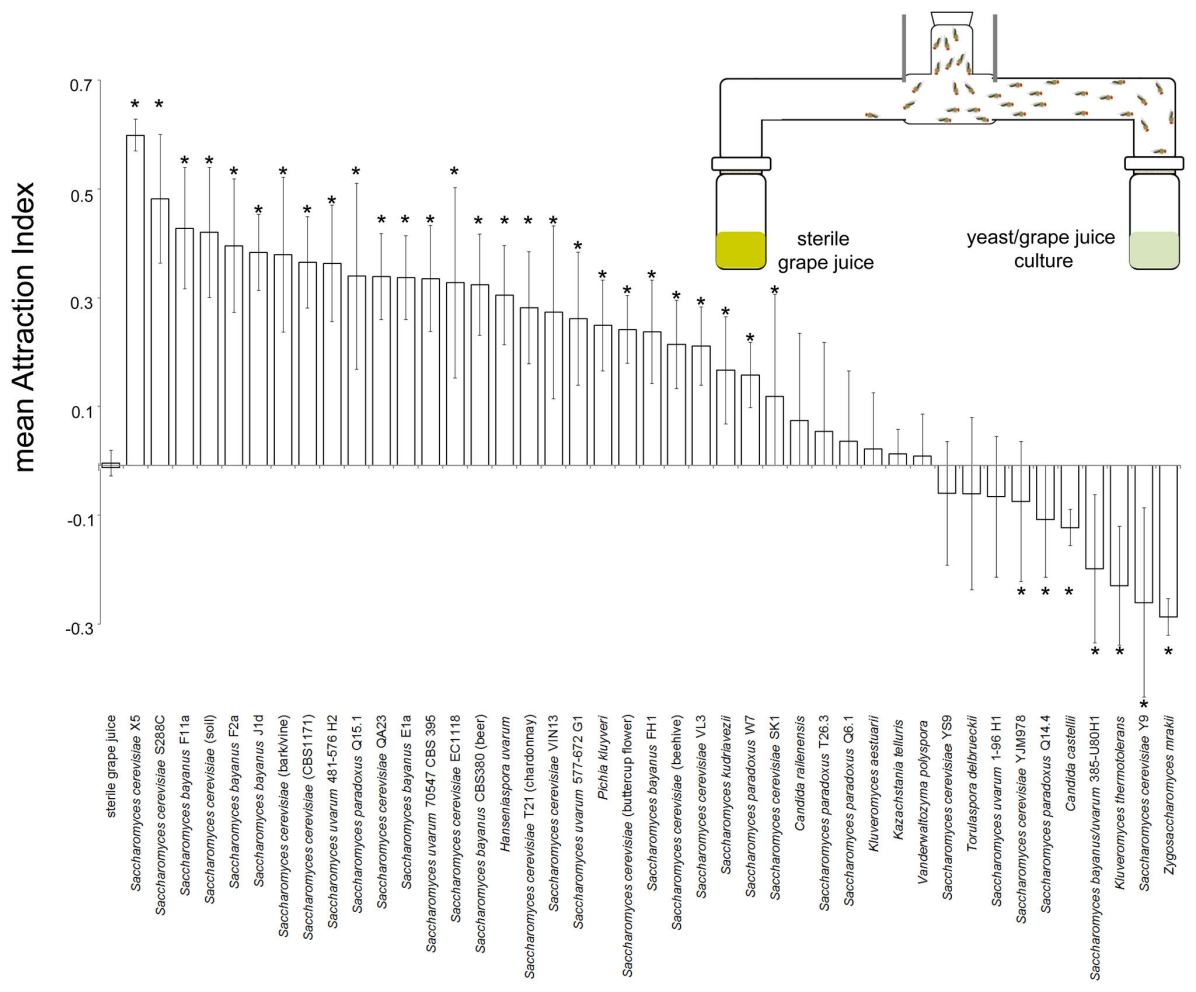
Illustration of T-maze apparatus and a plot of mean (±S.E. *n* = 8, in total an average of 343±30 (S.D.) fly choices were analysed per yeast strain) attraction index of female *Drosophila melanogaster* to a range of yeast isolates cultured in grape juice vs. sterile grape juice in paired choice tests. Asterisks above/below bars indicate a less than 5% probability that the flies had no preference given the observed total proportion of flies on either side of the arm, summed from all replicate T-maze tests per strain, and was calculated using the binominal distribution assuming an underlying 1:1 proportion.

The attractiveness of 43 genotypically distinct strains of yeast encompassing eight genera and 16 species, isolated from a variety of sources, were assayed in binary choice tests using adult female *D. melanogaster* (Figure 1 and Table S1). Each isolate was tested eight times against non-inoculated grape juice (control) and an attraction index (AI) calculated. Control juice v juice replicates produced an AI of 0.008 showing the flies had no preference for either side of the apparatus (*P* = 0.5, binomial), and the measurement standard error of the mean was small at just ~23% of the average experimental error (see Figure 1). AIs ranged from -0.28 to +0.61 and were approximately normally distributed (Shapiro-Wilk W=0.952, *P*=0.07). Flies were significantly attracted to 27 yeast isolates (mean±S.E. AI = 0.33±0.02; *P* < 0.05 with deviance from the null expectation of no preference estimated using the binomial distribution with the total number of flies that made a choice across all eight replicates, which averaged 343±30 (S.D); Figure 1 and Table S1). Flies were indifferent to nine isolates, (AI = 0.01±0.02, *P* > 0.05), and significantly repelled by seven isolates (AI = -0.18±0.03, *P* < 0.05). AIs were then normalized and arc-sine transformed for all subsequent statistical analyses. The variance in AI was unequal between isolates as revealed by an O’Brien test (F_[43,308]_ = 1.7013, *P* = 0.0059) and therefore we employed Welch’s ANOVA: this revealed that AIs were significantly different among isolates (F_[43,107]_ = 9.6649, *P* < 0.0001).

**Figure 2:**
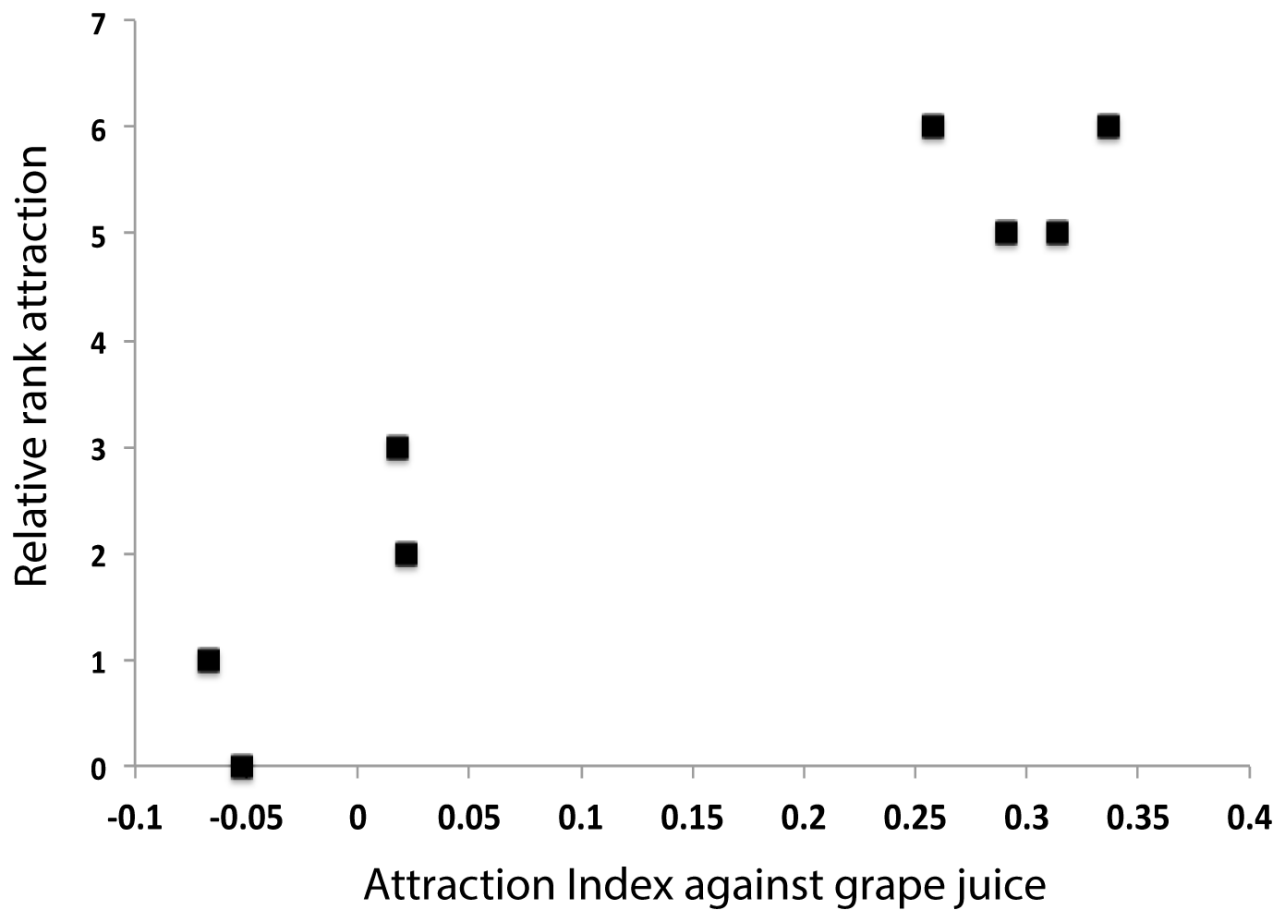
Attraction index (AI) of eight yeast isolates against grape juice correlated with the relative attraction index among the isolates from head-to-head comparisons. The relative attraction index is the number of times an isolate was more attractive to flies compared to the other seven isolates. These are significantly correlated, Pearson’s product-moment correlation = 0.95(*P* = 0.0004).

We chose four isolates that were significantly attractive ( *Pichia* *kluyveri* , *S. cerevisiae* T21, *Hanseniaspora* *uvarum* and *S. cerevisiae* EC118; *P* < 0.0001 by the binomial test), two to which the flies were indifferent ( *Kluyveromyces* *polysporus* and *Kazachstaniatelluris* ; *P* > 0.24, binomial) and two that were significantly repulsive (*S. cerevisiae* YJM978, *S. cerevisiae* YS9, *P* < 0.03, binomial). Relative rank attractiveness ascertained by 16 pairwise head-to-head competitions between all isolates were highly correlated with the strains’ AI (Pearson’s correlation = 0.95, *P* = 0.0004; Figure 2 and Table 1). Further, an alternative χ^2^ test comparing the null expectation of no association between head-to-head competitions and ranked attraction index revealed this association was highly significant (*P* = 0.00006). Therefore, our method to estimate attraction index appears robust and shows rankings are consistent among yeasts. We then evaluated two main factors to determine whether yeast taxonomic classification or source of isolation might be driving differences in AI.

**Table 1:** Attraction Indices (AI) of yeast isolates against grape juice compared to AI of head-to-head comparisons among isolates.

	AI (to Grape juice)	*P* *. kluyveri*	*H* *. uvarum*	*S. cerevisiae* YJM978	*S. cerevisiae* YS9	K. polysporus	A. telluris	*S. cerevisiae* EC1118
*P* *. kluyveri*	0.258							
*H* *. uvarum*	0.314	0.150						
*S. cerevisiae* YJM978	-0.067	-0.198	-0.206					
*S. cerevisiae* YS9	-0.052	-0.250	-0.445	-0.006				
K. polysporus	0.017	-0.310	-0.475	0.218	0.079			
A. telluris	0.022	-0.270	-0.412	0.014	0.010	-0.137		
*S. cerevisiae* EC1118	0.337	-0.012	0.160	0.289	0.432	0.318	0.261	
*S. cerevisae* Chardonnay	0.290	-0.202	0.398	0.209	0.298	0.282	0.362	-0.314

### Analyses of attraction by phylogenetic position

**Figure 3:**
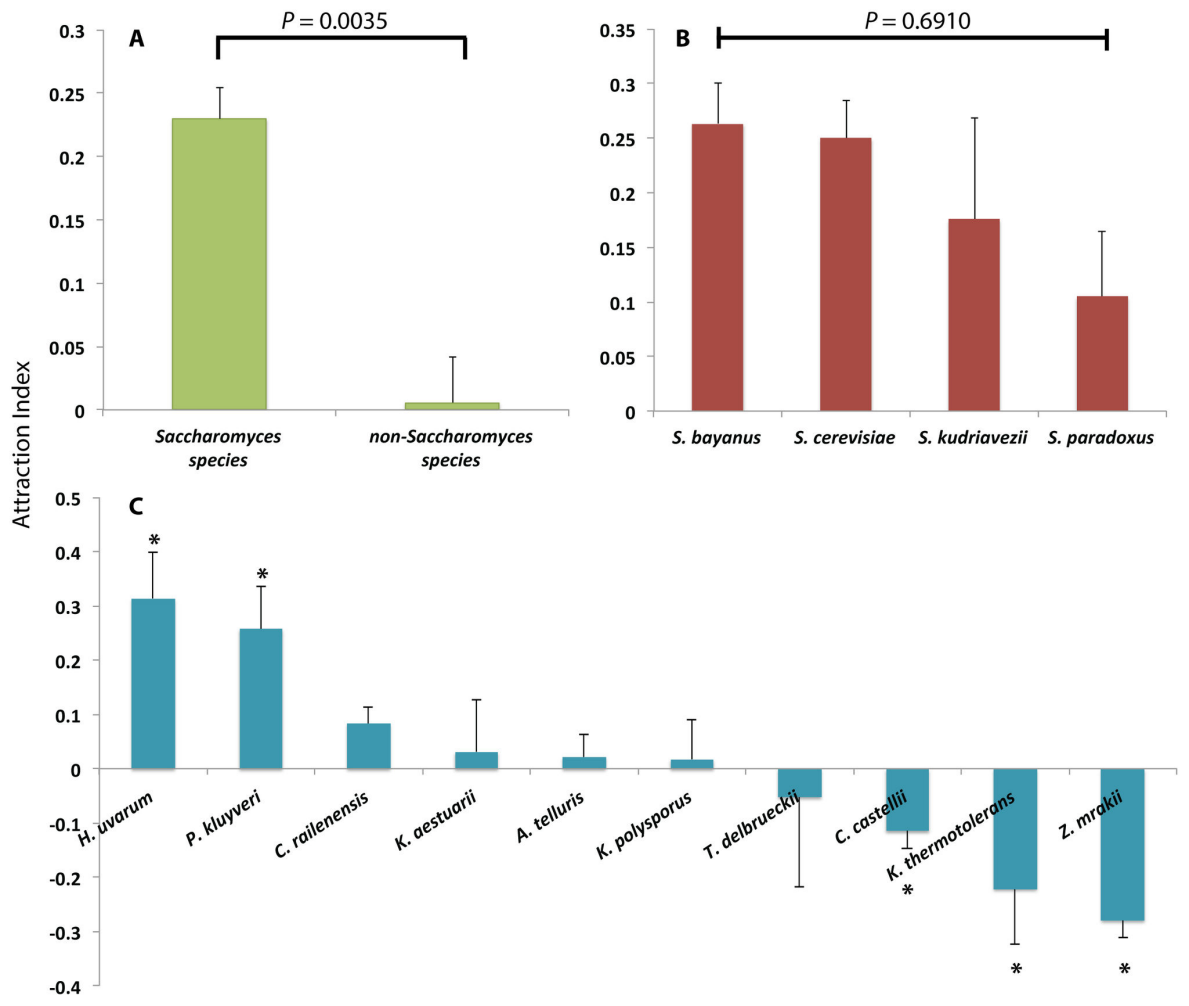
Comparisons of mean (±S.E) attraction indices among different levels of yeast taxonomic grouping. A: *Saccharomyces* isolates are significantly more attractive than non-*Saccharomyces* isolates (F_[1,41]_ = 9.5843, *P* = 0.0035). B: There is no significant difference in attraction among *Saccharomyces* species (F_[4,28]_ = 0.5636, *P* = 0.6910). C: There is significant difference in attraction among non-*Saccharomyces* species (F_[9,28]_ = 7.0988, *P* < 0.0001); *H* *. uvarum* and *P* *. kluyveri* were the only two significantly attractive non-*Saccharomyces* species. Asterisks above/below bars indicate a less than 5% probability that the flies had no preference given the observed total proportion of flies on either side of the arm, summed from all replicate T-maze tests per strain, and was calculated using the binominal distribution assuming an underlying 1:1 proportion.

Each isolate was first classified into either *Saccharomyces* (*n* = 33) or non- *Saccharomyces* (*n* = 10) genera, as this mainly splits these taxa into groups that are adapted to infest fruits via a strong Crabtree fermentation effect and those that are not [3,12-15,42]. The variances of these two groups were equal, and a mixed effects linear model showed the *Saccharomyces* genera were significantly more attractive (F_[1,41]_ = 9.5843, *P* = 0.0035; Figure 3A). We explored this taxonomic effect in finer detail and partitioned the genotypes by species within each of the *Saccharomyces* and non- *Saccharomyces* groups. The four *Saccharomyces* species we examined had equal variances (O’Brien test: F_[4,259]_ = 0.6653, *P* = 0.6166) and a mixed effects linear model revealed no significant difference in AI among *Saccharomyces* species for attractiveness (F_[4,28]_ = 0.5636, *P* = 0.6910; Figure 3B). The ten non- *Saccharomyces* species had unequal variances (O’Brien test: F_[9,70]_ = 3.0183, *P* = 0.0042) and a Welch’s ANOVA revealed significant differences among these species (F_[9,28]_ = 7.0988, *P* < 0.0001; Figure 3C). *H* *. uvarum* and *P* *. kluyveri* were the only two significantly attractive non- *Saccharomyces* species (*P* < 0.0001, binomial). Lastly, we tested for significant variance in attractiveness within *S. cerevisiae*, *S.* paradoxus and *S. bayanus/uvarum* since we have multiple isolates within these species. ANOVAs revealed no significant difference among *S. paradoxus* isolates (F_[4,35]_ = 1.6669, *P* = 0.1797), but revealed differences within *S. uvarum/bayanus* (F_[10,77]_ = 3.3041, *P* = 0.0013) and *S. cerevisiae* (F_[15,112]_ = 2.9364, *P* = 0.0006; Figure S1). One potential explanation for the difference among the *S. cerevisiae* (but not *S. uvarum/bayanus*) isolates is that it is driven by differences between the commercially available wine yeasts and ‘wild’ isolates. It seems reasonable to suggest that the wine industry will have selected and commercialized strains of yeast that produce the most appealing aromas during ferment, at least to humans, and that this might translate into altered volatile profiles that *Drosophila* can also differentiate. However, a mixed effects liner model revealed no difference (F_[1,14]_ = 0.7671, *P* = 0.3959) in attraction between the five commercially available winemaking strains compared to the eleven other *S. cerevisiae* isolates we assayed. Examining the variance within the *S. cerevisiae* population we evaluated, it appears the difference in attractiveness in this species is largely driven by the negative AIs of Y9, YJM978 and YS9 (Table S1 and Figures 1 & S1), which were respectively isolated from humans, rice ferments and used commercially for baking. These isolates are the only three *S. cerevisiae* that have mean negative AIs, and Y9 and YJM978 are significantly repulsive to *Drosophila* (*P* < 0.05, binomial). When these three strains are removed from the analyses, the data no longer support a significant difference in attractiveness among *S. cerevisiae* isolates (F_[12,91]_ = 1.008, *P* = 0.3693).

### Attraction by source of isolation

**Figure 4:**
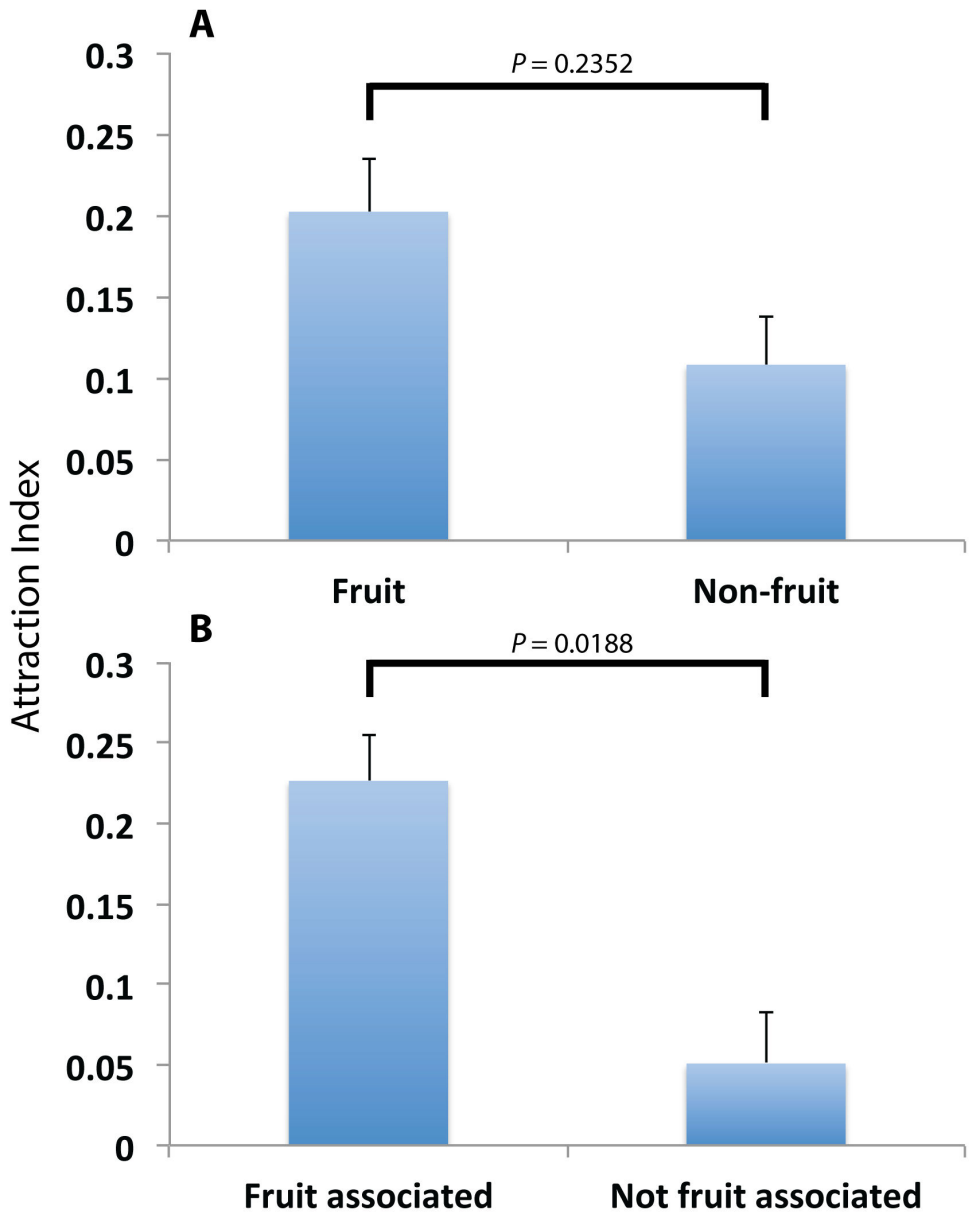
Comparisons of mean (±S.E) attraction indices when yeast isolates are partitioned according to their ecological origin. A: there is no significant difference in attraction according to whether yeast strains were directly isolated from fruits or not (F_[1,33]_ = 1.4618, *P* = 0.2352). B: there is a significant difference in attraction according to whether the strains were isolated from niches associated with fruit bearing plants or not (F_[1,33]_ = 6.1062, *P* = 0.0188).

While it seems that in general the *Saccharomyces* isolates are more attractive than the non- *Saccharomyces* ones, the observation that *S. cerevisiae* isolates derived from non-fruit associated niches repel *D. melanogaster*, and that non- *Saccharomyces* isolated from fruits/ferments are significantly attractive, suggests that the source of isolation might play a role in whether a yeast isolate is attractive or not. This is a less reliable factor to analyze as the isolation of a microbe from a certain location does not necessarily imply that this is the niche in which the strain normally resides: it might simply be a transient member of that particular microbial community. However, we next partitioned isolates as directly derived from fruit or non-fruit sources and ignored taxonomic classification (Figure 4A). For this separate analysis we removed commercial winemaking and baking strains, as well as isolates used in research, as they have been propagated, interbred and potentially developed in laboratories for extended periods since isolation. Analyses of the 35 ‘wild’ strains across the whole dataset with a mixed effects linear model revealed no significant difference in attraction according to whether strains were directly isolated from fruits or not (F_[1,33]_ = 1.4618, *P* = 0.2352; Figure 4A). However, if one chooses to partition the ‘wild’ isolates into those isolated from any vineyard or fruit niche, i.e. are associated with fruit-bearing plants (*n* = 20), or not (*n* = 15), then a mixed effects linear model revealed these groups significantly differ (F_[1,33]_ = 6.1062, *P* = 0.0188), with fruit associated isolates having an average increased (untransformed) AI of 0.18, see Figure 4B.

Finally, in a preliminary analysis, we investigated the nature of the odour blend as a factor in attraction. We were particularly interested in whether ethanol and acetic acid were driving these differences in the AI. Ethanol was detected by SPME analyses in the four yeast isolates chosen to span the range of AIs measured. However, ethanol was present in all strains, but an ANOVA revealed there was no relationship between ethanol, quantified as peak area in the total ion chromatogram (TIC), and attraction (F_[3,8]_ = 0.8934, *P* = 0.485). Acetic acid was not detected from any of the four isolates even though we could detect an acetic acid standard. Ethyl acetate was only detected in the two attractive strains, and of the eight volatile esters identified, only ethyl decanoate significantly differed among the strains tested (F_[3,8]_ = 10.4551, *P* = 0.0038, ANOVA), and further this was positively correlated with AI (F_[1,10]_ = 6.7830, *P* = 0.0263). From the analyses of TIC for a single replicate of each strain, we identified 82 different compounds in total, and 20, 34, 38, and 32 compounds for *K. telluris* *,* *P* *. kluyveri* , *S.* cerevisiae X5 and *S. cerevisiae* YJM978 respectively (see Figure S2 and Table S2). Except for *K* *. telluris* , of these volatiles, esters were the dominant chemical class identified, followed by alcohols. We predominantly detected alkanes from *K* *. telluris* , and this was the only strain from which we detected aldehydes (see Figure S2). We detected a greater range of acetate esters from *P* *. kluyveri* in comparison to the other strains tested. Of compounds implicated in attraction, acetoin (3-hydroxy butanone) was only detected in X5, and 3-methyl-1-butanol was present in both *S. cerevisiae* strains, and 2-phenyl ethanol was present in all strains except *K* *. telluris* . However, it is worth pointing out that the most and least attractive strains analysed (*S. cerevisiae* X5 and YJM978) had the greatest similarity of volatile profiles in total (Table S2).

## Discussion

Overall we found significant variance in *D. melanogaster* attraction among yeast isolates, with many yeast strains enhancing attraction over sterile grape juice. Attraction was strongly associated with the taxonomic classification of the strains of yeast. *Saccharomyces* species in general were more attractive to *Drosophila* . The strong association of *Saccharomyces* and *Drosophila* on decaying fruit is well documented [9,21], but the variance in attraction among differing yeasts has not been well characterised. Yeasts isolated from fruit and vineyard environments, regardless of taxonomic classification, were more attractive than yeasts isolated from non-fruit, non-vineyard sources.

We assayed the net attraction of *Drosophila* to various yeast isolates when inhabiting fruits, that is, all aspects of the ecology of an isolate that serve to attract flies. We did not measure change in yeast population densities, as population densities may not necessarily correlate with the ability of a yeast isolate to attract flies. The differences in attraction that we observed are reasonably due to differences in the mix of aroma compounds produced by the different yeasts isolates. We show that differential attraction is not likely due to ethanol or acetic acid production, despite the known preferences of *D. melanogaster* for these compounds [9,24,38]. Other work shows that a mixture of volatile compounds most strongly elicits fly attraction and a blend of both fruit and yeast odours are required to stimulate female *Drosophila* oviposition [16-18,24,41], and our data tend to agree with the previous volatile compounds implicated. Thus, it is likely some other compounds in the blend here contribute to attraction, possibly including ethyl deconate.

The attraction of *Drosophila* to only some yeast isolates is suggestive of, or at the least provides the potential for, co-evolution between these flies and certain yeasts, particularly *Saccharomyces* , and/or species that are associated with fruiting plants generally (in this case *Pichia* and *Hansenispora* species). While we have not conclusively shown which compounds are responsible, the design of the choice chambers in this study strongly suggests that volatiles produced by these yeasts serve to attract flies. This observation fits with the hypothesis suggesting that at least one biological function of volatile production by yeasts is to attract insect vectors. Such attraction presumably enhances the likelihood of dispersal for yeasts inhabiting the ephemeral fruits. Not only is dispersal an important component of fitness for sessile organisms, but dispersal via the gut of *Drosophila* likely increases the rates of sexual reproduction and outcrossing [18-20], and thus genetic diversity and rates of adaptation [21-23,43,44]. Indeed outcrossed matings are inferred to be more common in fruit-dwelling *S. cerevisiae* compared to the similar but non-fruit dwelling congener *S. paradoxus* [24,45,46].

Our data show exceptions to the rule of *Saccharomyces* being most attractive to *Drosophila* , as some non- *Saccharomyces* isolates are at least equally as attractive and some *Saccharomyces* isolates are repulsive. Two weakly or non-fermenting yeasts collected from fruits, *Hanseniasporum* *uvarum* and *Pichia* *kluyveri* , were also highly attractive to *D. melanogaster*. Other studies reveal these species as being associated with *Drosophila* [19,23,39,40], and certain isolates may produce volatiles potentially attractive to *Drosophila* [13,20,23-25]. The fact that some weakly or non-fermenting yeasts are attractive to flies suggests attraction is not simply correlated with the strongly Crabtree positive *Saccharomyces* complex, but may evolve independently of fermentation. In comparison, the non-attractive *S. cerevisiae* are non-fruit derived with the four repulsive *Saccharomyces* species isolates deriving from humans, rice ferments, oak bark and commercial baking (Figure 1). Together this hints at attractiveness being a labile trait, which can be gained and lost both within and between species, possibly in response to the habitat differences. Another alternative is that volatile production evolved for reasons not linked to insect attraction (detoxifying ferment intermediates), and flies secondarily evolved to follow these volatile cues. Attractive volatile production may have been lost in *Saccharomyces* lineages no longer associated with fruits, and it is possible that weakly or non-fermenting non- *Saccharomyces* inhabiting fruits evolved the ability to mimic attractiveness, despite not harbouring an ability to alter the niche via fermentation.

It has been suggested that *Drosophila* influence the density and community structure of yeasts on fruit to their benefit through a process of ecosystem engineering [16,18,22,39]. However, perhaps yeasts through the differential production of volatiles also manipulate this ecosystem by attracting certain *Drosophila* which enhances their ability to disperse and outcross. Ecosystem engineering by species such as *S. cerevisiae* is typically associated with competition and exclusion of other microbes [26,47-49]. But perhaps ecosystem engineering can also evolve into a mutualism, particularly when two organisms are engineering both the niche and thus each other indirectly.

## Materials and Methods

### Culturing

*Drosophila melanogaster* were trapped in the field (Auckland, New Zealand), identified according to [27,28,50] and maintained at 25°C and ~70% relative humidity in a 12h light: 12h dark photoperiod on Formula4-24 Instant *Drosophila* *medium* without addition of yeast (Carolina Biological Supply). Flies were sex sorted after 2-3 days and used within 4 days. Yeasts were grown in YPD (10 g yeast extract, 20 g peptone, 20 g glucose, 15g agar, 1L distilled water) at 28°C for two days, and then maintained at 4°C. Yeasts were also stored in 15% glycerol at -80°C. Ferments were prepared with 50 mL of sterilised 2008 Sauvignon blanc grape juice sourced from Marlborough, New Zealand. Grape juice was sterilised at 25°C for 24 h by adding 1mL/L of Dimethly Dicarbonate (DMDC, in ethanol 1:4), an odourless and easily degradable steriliser regularly used in winemaking [7,9,12,29,30,51]. Ferments for the behavioural assays and scent analyses were initiated with 10^5^ yeast cells pre-cultured in YPD broth at 25°C, 120 rpm for 2 days, added to 50 mL of sterilized grape juice and cultured for a further 2 days. Cultures and grape juice controls were diluted to 1:1000 with sterile water before use in trials.

### Behavioural assays testing yeast attractiveness

Behavioural assays to test the preferences of *Drosophila melanogaster* among yeast isolates were conducted using a two-choice T-maze apparatus (Figure 1). The two arms of the T-maze were loaded with 50 mL of diluted sterile grape juice (control) vs. 50 mL of a diluted grape juice (experimental) cultured with one of 43 yeast isolates (Table 1). A second series of trials involved comparison of grape juices with one of two different yeast strains, one in each arm of the T-maze. Each trial involved 70-80, 3-7 day old female flies, deprived of food and water for 9 hours (*n* = 8 replicates per yeast isolate) and briefly anaesthetised by cooling at 4°C before being loaded into the entry tube of the T-maze. After 30 minutes in the dark, the two arms of the T-maze were blocked, the apparatus was frozen to euthanize the flies, and the number of flies present in the arms and entry tube were counted. The relative attractiveness of the yeast was calculated as: (flies in experimental arm – flies in control arm)/(total flies in experiment – flies remaining in entry tube), adapted from [52,53]. T-mazes were thoroughly washed and air-dried between trials. The binomial distribution was employed to determine the significance of fly choice preference: data were pooled across the eight replicates and the number of flies that made a choice either way were compared to the null expectation of equal proportions in each arm of the T-maze.

For analyses requiring the grouping of strains, variance in the data were analyzed using mixed effect models, and isolate was treated as a random effect (*n*=8), and the factor of interest (taxonomic classification or source of isolation) as the fixed effect. Tests between individual strains were analysed with standard ANOVA. Tests for equal variances were conducted in all cases and Welch’s tests employed if the assumption of equal variances could not be satisfied, and ANOVAs used wherever possible. All tests were conducted with JMP v10 (SAS).

### Volatile analyses

Yeast cultures were prepared as in the behavioural assays except that a 1:1000 dilution was not performed. 10 mL of each culture was sealed in glass vials with septa made from low-volatile polyester oven bags and volatile odours were allowed to equilibrate for 30 min. The headspace was sampled for an additional 30 mins by SPME (solid phase microextraction; Supelco 65µm PDMS-DVB). Volatiles were analysed by gas chromatography-mass spectrometry (GC-MS) on an Agilent 7890A GC coupled with a 5975 MS. The injector temperature was 260°C, the oven was held at 40°C for 4 min, increased at 5 °C/min to 100°C, then increased at 10 °C/min to 220°C and held for 9 min [54]. The column was an Agilent DB-1701 (30m x 25µm x 0.15µm) and the carrier gas was nitrogen. Compounds were identified by comparison with the MS library (NIST 05) and injection of synthetic standards for ethanol, acetic acids and eight esters. In addition, the complete total ion chromatogram for one replicate of each strain was analysed, with every peak identified by comparison with the NIST 05 library using AMDIS_32 (http://www.amdis.net), see Table S2.

## Supporting Information

Table S1 **Yeasts tested for attractiveness to female *Drosophila melanogaster* in choice assays in comparison to sterile grape juice.**
(DOCX)

Table S2 **Presence of compounds ascertained by analysing the total ion chromatogram of SPME captured headspace volatiles for one replicate culture of each yeast strain.** Every peak in the ion chromatogram was identified by comparison with the NIST 05 library using AMDIS_32 (http://www.amdis.net).
(DOC)

Figure S1 **Mean (±S.E., *n* = 8) Attraction Indices (AI) of individual *S. cerevisiae*, *S.* bayanus and *S. paradoxus* isolates.**
(TIF)

Figure S2 **Breakdown of major classes of volatiles detected by analysing the total ion chromatogram of SPME captured headspace volatiles for one replicate culture of each yeast strain.** Every peak in the ion chromatogram was identified by comparison with the NIST 05 library using AMDIS 32 (http://www.amdis.net). The attractivness of each yeast strain is indicated - compare to Figure 1and Table S2.
(TIF)
