# Supplementary material for: Quantifying Variation in the Ability of Yeasts to Attract Drosophila melanogaster
Source: PLoS One. 2013 Sep 25;8(9):e75332. doi: 10.1371/journal.pone.0075332 (PMC3783394; doi:10.1371/journal.pone.0075332)
Supplement: Table S1 — Yeasts tested for attractiveness to female Drosophila melanogaster in choice assays in comparison to sterile grape juice. (DOCX) [file pone.0075332.s001.docx]

| **Yeast species and strain** | **Origin** | **Reference** | **Fly preference vs. sterile grape juice** | |
| --- | --- | --- | --- | --- |
|  |  |  | **AI*** | **P^†^** |
| *Grape juice/wine ferment isolates:* | |  |  |  |
| *Candida railenensis* | Chardonnay juice (West Auckland) | [1] | 0.08 | 0.21 |
| *Hanseniaspora uvarum* | Chardonnay juice (Gisborne) | Goddard lab collection | 0.31 | < 0.01 |
| *Pichia kluyveri* | Chardonnay juice (West Auckland) | [1] | 0.26 | < 0.01 |
| *Saccharomyces bayanus* E1a | Sauvignon Blanc ferment (West Auckland) | Goddard lab collection | 0.35 | < 0.01 |
| *S. bayanus* F11a | Sauvignon Blanc ferment (West Auckland) | Goddard lab collection | 0.44 | < 0.01 |
| *S. bayanus* F2a | Sauvignon Blanc ferment (West Auckland) | Goddard lab collection | 0.40 | < 0.01 |
| *S. bayanus* FH1 | Sauvignon Blanc ferment (West Auckland) | Goddard lab collection | 0.25 | < 0.01 |
| *S. bayanus* J1d | Sauvignon Blanc ferment (West Auckland) | Goddard lab collection | 0.39 | < 0.01 |
| *S. cerevisiae* T21 | Chardonnay ferment (West Auckland) | [2] | 0.29 | < 0.01 |
| *S.bayanas/uvarum* 385-U80H1 | Sauvignon blanc juice (Marlborough) | Goddard lab collection | -0.19 | < 0.01 |
| *S. uvarum* 1-96 H1 | Sauvignon blanc juice (Marlborough) | Goddard lab collection | -0.06 | 0.21 |
| *S. uvarum* 481-576 | Sauvignon blanc juice(Marlborough) | Goddard lab collection | 0.37 | < 0.01 |
| *S. uvarum* 577-672 G1 | Sauvignon blanc juice(Marlborough) | Goddard lab collection | 0.27 | < 0.01 |
| *Torulaspora delbrueckii* | Chardonnay juice (Gisborne) | Goddard lab collection | -0.05 | 0.15 |
| *Vineyard isolates:* | |  |  |  |
| *Kluyveromyces thermotolerans* | Sauvignon blanc fruit (Marlborough) | Goddard lab collection | -0.22 | < 0.01 |
| *S. cerevisiae* B161105841-2 | Beehive (West Auckland) | [2] | 0.22 | < 0.01 |
| *S. cerevisiae* MG161105S24-30 | Chardonnay vine bark (West Auckland) | [2] | 0.39 | < 0.01 |
| *S. cerevisiae* MG311005S14-1 | Vineyard soil (West Auckland) | [2] | 0.43 | < 0.01 |
| *S. cerevisiae* Mi161105S32-3 | Buttercup located in vineyard (West Auckland) | [2] | 0.25 | < 0.01 |
| *Non-‘vineyard/fruit’ isolates:* | |  |  |  |
| *S. paradoxus* Q14.4 | Oak tree, Windsor Great Park, UK | [3] | -0.10 | < 0.05 |
| *S. paradoxus* Q15.1 | Oak tree, Windsor Great Park, UK | [3] | 0.35 | < 0.01 |
| *S. paradoxus* Q6.1 | Oak tree, Windsor Great Park, UK | [3] | 0.05 | 0.22 |
| *S. paradoxus* T26.3 | Oak tree, Silwood Park, UK | [3] | 0.06 | 0.48 |
| *S. paradoxus* W7 | Oak tree, Silwood Park, UK | [3] | 0.17 | < 0.05 |
| *Zygosaccharomyces mrakii* | CBS 4218, Silage, Italy | www.cbs.knaw.nl | -0.28 | < 0.01 |
| *Candida castellii* | CBS 4332, Soil, Finland | www.cbs.knaw.nl | -0.11 | 0.20 |
| *Kazachstania telluris* (prev. *Arxiozyma telluris*) | CBS 2685, Soil South Africa. | www.cbs.knaw.nl | 0.02 | 0.38 |
| *Kluyveromyces aestuarii* | CBS 4438, Estuary mud, Brazil | www.cbs.knaw.nl | 0.03 | 0.25 |
| *Vanderwaltozyma polyspora* (prev. *K. polysporous*) | CBS 2163, Soil, South Africa | www.cbs.knaw.nl | 0.02 | 0.55 |
| *S. bayanus* | AWRI 1146, CBS 380, isolated from beer. | www.cbs.knaw.nl | 0.33 | < 0.01 |
| *S. cerevisiae* | AWRI 74, CBS 1171, brewer’s yeast, Oranjeboom brewery, Netherlands | www.cbs.knaw.nl | 0.37 | <0.01 |
| *S. cerevisiae* Y9 | From Indonesian ragi (yeast cake), made by fermenting koji (rice plus *Aspergillus oryzae*). | [4] | -0.25 | < 0.01 |
| *S. kudriavezii* | CBS 8840, from decaying leaves | www.cbs.knaw.nl | 0.22 | < 0.01 |
| *S. cerevisiae* YJM978 | Vaginal isolate | [5] | -0.07 | 0.035 |
| *Commercial and Research strains:* | |  |  |  |
| *Saccharomyces cerevisiae* EC1118 | Wine - Lalvin (Lallemand) | www.lallemand.com | 0.34 | < 0.01 |
| *S. cerevisiae* QA23 | Wine - Lalvin (Lallemand) | www.lallemand.com | 0.35 | < 0.01 |
| *S. cerevisiae* VIN13 | Wine - Anchor (Lallemand) | www.lallemand.com | 0.28 | < 0.01 |
| *S. cerevisiae* VL3 | Wine - Zymaflore (Laffort) | www.laffort.com | 0.22 | < 0.01 |
| *S. cerevisiae* X5 | Wine - Zymaflore (Laffort) | www.laffort.com | 0.61 | < 0.01 |
| *S. cerevisiae* YS9 | Baker’s yeast (Le Saffre trade name), Singapore | [4] | -0.05 | 0.099 |
| *S. cerevisiae* SK1 | Research strain | [4] | 0.13 | < 0.01 |
| *S. cerevisiae* S288C | Research strain | [4] | 0.49 | < 0.01 |

**Table S1.** Yeasts tested for attractiveness to female *Drosophila melanogaster* in choice assays in comparison to sterile grape juice.

AWRI: Australian Wine Research Institute, Urrbrae, South Australia. CBS: Centraalbureau voor Schimmelcultures, Utrecht, The Netherlands. Anchor and Lalvin yeasts owned by Lallemand, Montreal, Canada. Zymaflore yeasts: Laffort, Bordeaux, France.

*Mean attractiveness in grape juice ferments in comparison to sterile Sauvignon blanc grape juice when tested with a two-choice T-maze apparatus (*n* = 8 replicates per yeast variety, each involving ~80 female *Drosophila melanogaster*). Higher positive values indicate stronger attraction to yeast ferment over sterile grape juice. Negative values indicate repulsion. ^†^Binomial probability of the observed distribution of flies that made a choice compared to the null expectation of no choice preference (i.e. a 1:1 distribution).

1. Goddard MR (2008) Quantifying the complexities of Saccharomyces cerevisiae's ecosystem engineering via fermentation. Ecology 89: 2077–2082.

2. Goddard MR, Anfang N, Tang R, Gardner RC, Jun C (2010) A distinct population of Saccharomyces cerevisiae in New Zealand: evidence for local dispersal by insects and human-aided global dispersal in oak barrels. Environ Microbiol 12: 63–73. doi:10.1111/j.1462-2920.2009.02035.x.

3. Johnson LJ, Koufopanou V, Goddard MR, Hetherington R, Schäfer SM, et al. (2004) Population genetics of the wild yeast Saccharomyces paradoxus. Genetics 166: 43–52.

4. Liti G, Carter DM, Moses AM, Warringer J, Parts L, et al. (2009) Population genomics of domestic and wild yeasts. Nature 458: 337–341. doi:10.1038/nature07743.

5. Schacherer J, Shapiro JA, Ruderfer DM, Kruglyak L (2009) Comprehensive polymorphism survey elucidates population structure of Saccharomyces cerevisiae. Nature 458: 342–345. doi:10.1038/nature07670.
