# Supplementary figures and images for: Quantifying Variation in the Ability of Yeasts to Attract Drosophila melanogaster
Source: PLoS One. 2013 Sep 25;8(9):e75332. doi: 10.1371/journal.pone.0075332 (PMC3783394; doi:10.1371/journal.pone.0075332)

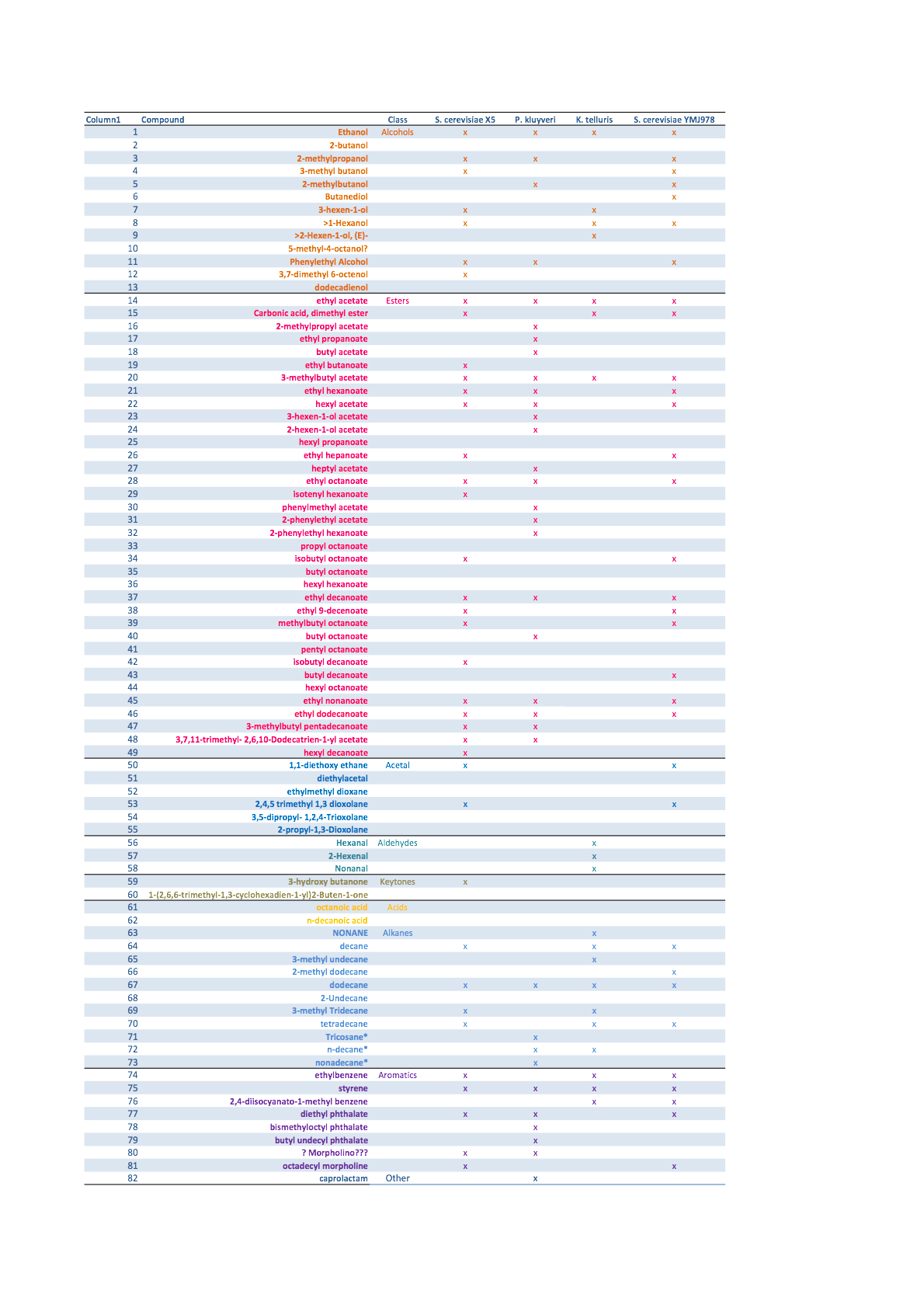

Supplement: Table S2 — Presence of compounds ascertained by analysing the total ion chromatogram of SPME captured headspace volatiles for one replicate culture of each yeast strain. Every peak in the ion chromatogram was identified by comparison with the NIST 05 library using AMDIS_32 (http://www.amdis.net). (DOC) [file pone.0075332.s002.doc]

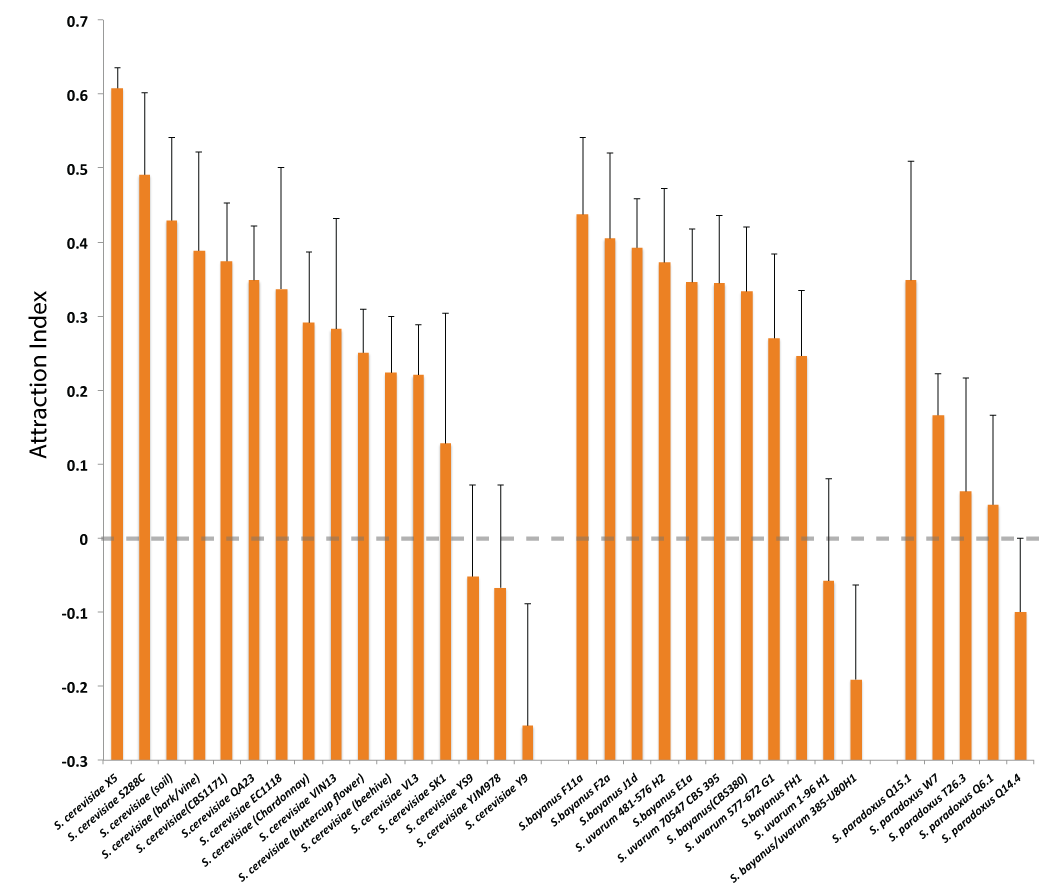

Supplement: Figure S1 — Mean (±S.E., n = 8) Attraction Indices (AI) of individual S. cerevisiae, S. bayanus and S. paradoxus isolates. (TIF) [file pone.0075332.s003.tif]

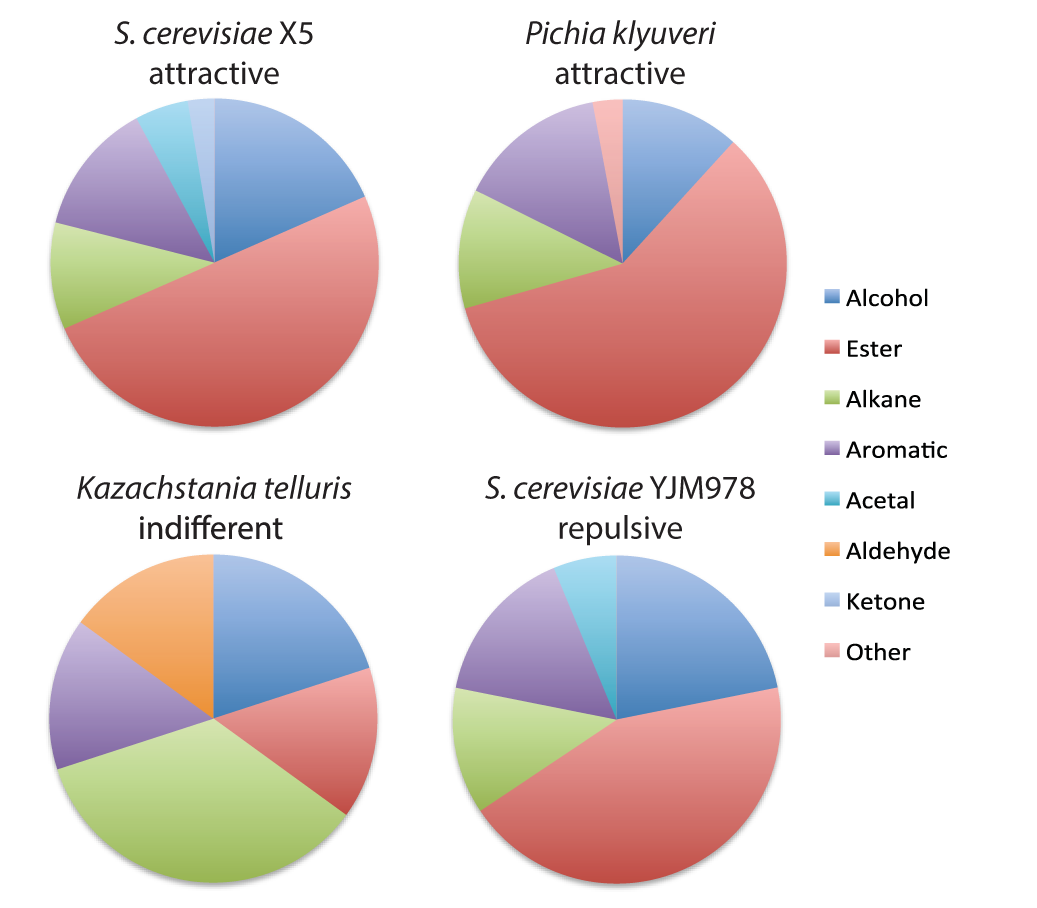

Supplement: Figure S2 — Breakdown of major classes of volatiles detected by analysing the total ion chromatogram of SPME captured headspace volatiles for one replicate culture of each yeast strain. Every peak in the ion chromatogram was identified by comparison with the NIST 05 library using AMDIS 32 (http://www.amdis.net). The attractivness of each yeast strain is indicated - compare to Figure 1and Table S2. (TIF) [file pone.0075332.s004.tif]
